# Decreased quadriceps muscle stiffness on ultrasound elastography is associated with sarcopenia in end-stage kidney disease

**DOI:** 10.3389/fneph.2025.1682826

**Published:** 2025-12-11

**Authors:** Chien-Hua Chiu, Jia-Feng Chen, Shan-Fu Yu, Chung-Yuan Hsu, Ying-Chou Chen

**Affiliations:** 1 Department of Nephrology, Kaohsiung Chang Gung Memorial Hospital, Chang Gung University College of Medicine, Kaohsiung, Taiwan; 2 Department of Rheumatology, Kaohsiung Chang Gung Memorial Hospital, Kaohsiung, Taiwan; 3 Department of Rheumatology, Kaohsiung Municipal Ta-Tung Hospital, Kaohsiung, Taiwan

**Keywords:** end-stage kidney disease (ESKD), sarcopenia, ultrasound sonoelastography, dual-energy X-ray absorptiometry (DXA), quadriceps

## Abstract

**Background:**

Sarcopenia has emerged as one of the major complications in end-stage kidney disease (ESKD), leading to greater disability and poor long-term outcomes. This study aimed to compare quadriceps muscle sonoelastographic parameters between ESKD patients with and without sarcopenia.

**Materials and methods:**

We prospectively enrolled 50 ESKD patients with sarcopenia and 50 ESKD patients without sarcopenia as controls. All participants underwent clinical and laboratory evaluation, sonoelastography of the quadriceps muscle, and dual-energy X-ray absorptiometry (DXA) for muscle mass assessment. Sarcopenia was diagnosed according to the revised European Working Group on Sarcopenia in Older People (EWGSOP2, 2019), which emphasizes muscle strength as the principal determinant. Handgrip strength, gait speed, and appendicular skeletal muscle mass (ASM/height²) by DXA were assessed. The elastography ratio was calculated as the stiffness of the quadriceps muscle relative to the overlying subcutaneous tissue. Comparisons were made between the sarcopenia and non-sarcopenia groups.

**Results:**

A total of 100 ESKD patients were included: 50 with sarcopenia (mean age, 63.0 ± 12.7 years) and 50 without sarcopenia (mean age, 58.3 ± 14.9 years). The sarcopenia group demonstrated a lower quadriceps-to-subcutaneous tissue elastography ratio compared with the control group. Multivariate logistic regression identified the quadriceps-to-subcutaneous tissue ratio, muscle hardness, and body mass index (BMI) as independent predictors of sarcopenia (p < 0.05). Lower BMI was associated with an increased risk of sarcopenia. The optimal quadriceps-to-subcutaneous tissue elastography ratio cut-off value was 0.885 (sensitivity 82.4%; specificity 66.7%).

**Conclusion:**

Sonoelastography provides a reliable and non-invasive assessment of quadriceps muscle stiffness and demonstrates good predictive value for detecting sarcopenia in ESKD patients. Given its accessibility, low cost, and ease of use, sonoelastography may serve as a valuable adjunct to conventional DXA in evaluating muscle quality in this high-risk population.

## Introduction

The global population is aging, highlighting the importance of understanding degenerative processes that accompany advancing age (1). One such process is sarcopenia, defined as the progressive, age-related loss of skeletal muscle mass, strength, and physical performance (2). Sarcopenia is associated with adverse health outcomes, including impaired mobility, increased risk of falls and fractures, difficulty performing activities of daily living, disability, loss of independence, and increased mortality death (3–5).

The etiology of sarcopenia is multifactorial, involving environmental influences, chronic diseases, systemic inflammation, physical inactivity, mitochondrial dysfunction, neuromuscular junction degeneration, depletion of satellite cells, and hormonal alterations (6). Sarcopenia is particularly prevalent in patients with chronic kidney disease (CKD) (7–9), with estimates suggesting that approximately 37% of dialysis patients are affected (10). In this population, muscle loss is strongly linked to increased morbidity and mortality, especially due to cardiovascular complications (11, 12). Early detection and assessment of modifiable risk factors are therefore crucial.

While most studies focus on sarcopenia in patients with CKD receiving dialysis, fewer have investigated sarcopenia in those with end-stage kidney disease (ESKD) who are not yet on dialysis (13, 14). Identifying reliable, non-invasive assessment tools in this population may help improve early diagnosis and intervention.

Sonoelastography—first introduced by Ophir et al.—is an imaging technique that measures the deformation of tissue in response to external force applied by an ultrasound transducer (13). Tissue stiffness varies depending on its structural composition: soft tissues deform more readily, whereas stiffer tissues deform less (14, 15). This mechanical property is displayed on the ultrasound monitor as a color spectrum, where blue typically denotes stiff regions, red represents soft regions, and green indicates intermediate stiffness (16). Initially applied to differentiate benign from malignant tumors, sonoelastography is most commonly used in breast and thyroid imaging. Its use in the musculoskeletal system is relatively recent but is gaining interest (17–19).

In musculoskeletal evaluation, sonographic assessment of the quadriceps muscle is often one of the first imaging approaches, complementing clinical examination and radiography. However, to date, no published studies have specifically examined quadriceps muscle stiffness using sonoelastography in ESKD patients with sarcopenia.

Therefore, the present study aimed to compare quadriceps muscle sonoelastographic findings between ESKD patients with and without sarcopenia, and to explore the potential role of sonoelastography in the early detection of sarcopenia in this high-risk population.

## Materials and methods

### Study design

This was a case–control study conducted over a one-year period. The case group consisted of patients diagnosed with end-stage kidney disease (ESKD) and sarcopenia, while the control group included ESKD patients without sarcopenia.

### Patient selection

#### Diagnostic criteria for ESKD

ESKD was diagnosed according to established guidelines (22), based on evidence of kidney damage and/or reduced kidney function as determined by glomerular filtration rate (GFR), irrespective of the underlying cause.

#### Diagnostic criteria for sarcopenia (EWGSOP2, 2019)

Sarcopenia was diagnosed according to the updated EWGSOP2 guidelines. The diagnostic process followed a hierarchical approach emphasizing muscle strength as the key criterion:

1. Muscle Strength: Handgrip strength was measured using a calibrated Jamar dynamometer in the dominant hand. Low muscle strength was defined as <27 kg for men and <16 kg for women.
2. Muscle Mass: Appendicular skeletal muscle mass (ASM) was measured by dual-energy X-ray absorptiometry (DXA). Low muscle mass was defined as ASM/height² <7.0 kg/m² for men and <5.5 kg/m² for women.
3. Physical Performance: Gait speed was evaluated over a 4-meter walk at usual pace. Low physical performance was defined as gait speed ≤0.8 m/s.

Sarcopenia was confirmed when both low muscle strength and low muscle mass were present. Severe sarcopenia was diagnosed when all three components were reduced.

Patients were reclassified according to EWGSOP2 criteria. After reanalysis, 48 patients fulfilled the definition of sarcopenia and 52 were classified as non-sarcopenic. The distribution was comparable to the previous EWGSOP (2010) classification due to consistent overlap of low strength and low mass in this advanced ESKD cohort.

According to EWGSOP2 (2019) criteria, 48 patients were classified as sarcopenic and 52 as non-sarcopenic. Baseline demographic and clinical characteristics were similar to the previous analysis. The sarcopenia group demonstrated significantly lower quadriceps-to-subcutaneous tissue elastography ratios compared with the control group. Multivariate logistic regression confirmed that lower elastography ratio, reduced muscle hardness, and lower BMI remained independent predictors of sarcopenia (p < 0.05). The optimal cut-off value for the quadriceps-to-subcutaneous ratio was 0.885 (sensitivity 82.4%; 1-specificity 33.3%).

#### Inclusion criteria

• Patients aged 20–70 years with a confirmed diagnosis of ESKD.

#### Exclusion criteria

Patients were excluded if they had any of the following:

1. Active infection.
2. History of primary intracerebral hemorrhage, myocardial infarction, unstable symptomatic peripheral vascular disease, major surgery, or systemic hemorrhage within the previous 3 months.
3. Known malignancy or hematologic disorders affecting thrombosis, platelet count, or function.
4. Severe systemic disease such as liver cirrhosis or congestive heart failure.

### Clinical and laboratory assessment

Baseline demographic and clinical data, including age, sex, symptom duration, and current medications, were recorded. Laboratory assessments were performed according to standard protocols.

### Muscle mass assessment by DXA

Muscle mass was measured using dual-energy X-ray absorptiometry (DXA). Low muscle mass was defined as an appendicular skeletal muscle mass index (ASM/height²) more than two standard deviations below the mean value of a healthy young reference population (<7.23 kg/m² for men and <5.67 kg/m² for women).

### Ultrasound sonoelastography

Sonoelastographic measurements of the quadriceps muscle were performed using a high-frequency linear transducer. Quantitative values were obtained from circular regions of interest placed within the quadriceps muscle. Images were acquired in the longitudinal plane during the compression phase. Conventional B-mode images were displayed on the left side of the screen, and color-coded sonoelastographic maps on the right. Tissue stiffness was represented by a standardized color scale, and elastography ratios were calculated as the stiffness of the quadriceps muscle relative to the overlying subcutaneous tissue (20).

### Statistical analysis

The statistical analysis was conducted by using SPSS version 22 software (IBM Corporation, Armonk, NY). Multivariate regressions and T-tests were utilized to compare difference and correlation of sonoelastography and dual energy X-ray absorptimetry assessments in ESKD with sarcopenia. Significance levels were set at P <.05.

## Results

**Figure 1:**
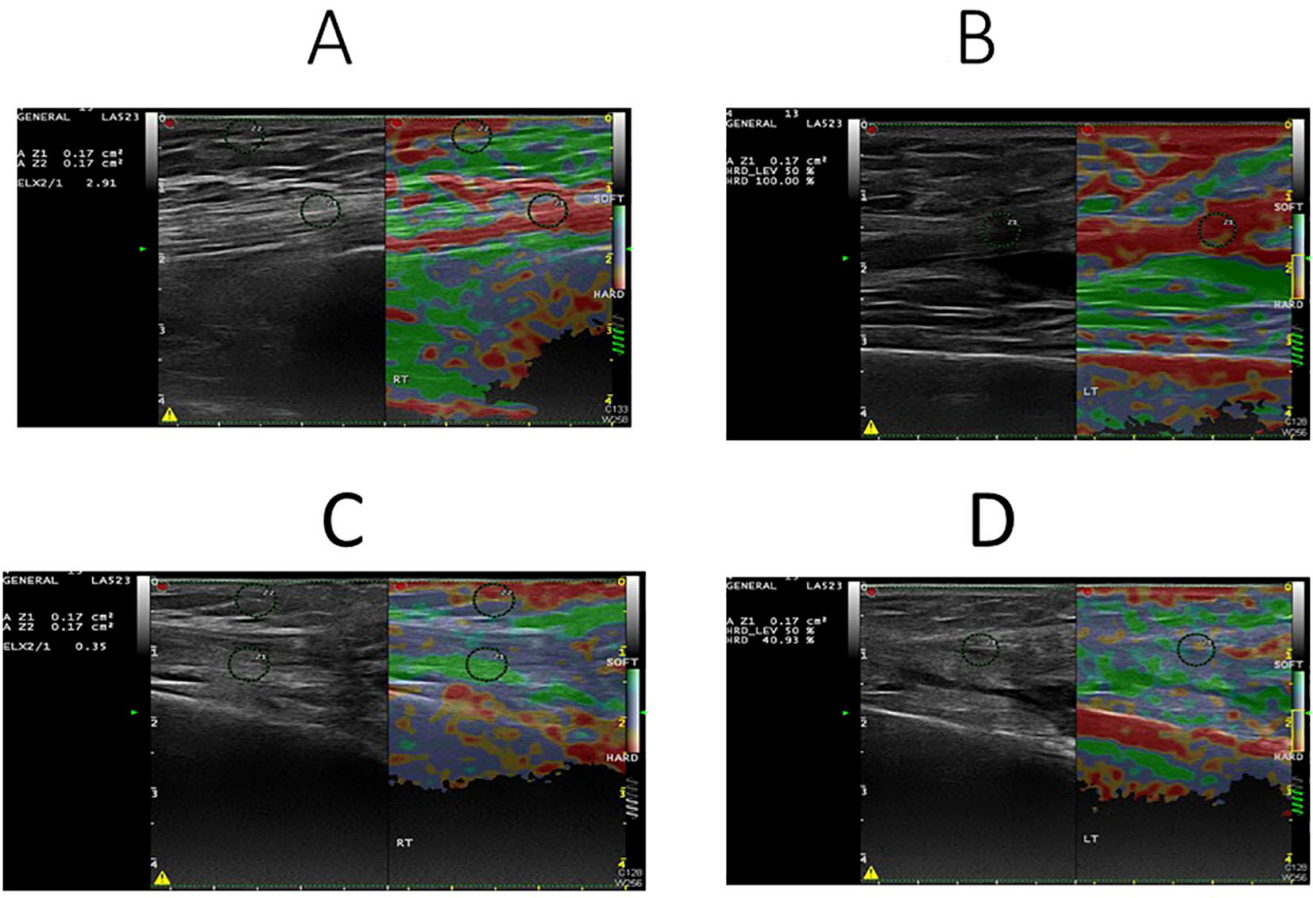
Sonoelastography images of quadriceps muscle in normal **(A, B)** and sarcopenic **(C, D)** patients. The elasticity ratio of quadriceps muscle to subcutaneous fat was 2.91 in the normal patient **(A)** and 0.35 in the sarcopenic patient **(C)**. Correspondingly, muscle hardness was 100% in the normal patient **(B)** and 40.93% in the sarcopenic patient **(D)**.

A total of 100 ESKD patients including 50 with sarcopenia (mean age 63.02 ± 12.72 years) and 50 without sarcopenia (mean age 58.30 ± 14.936 years) were enrolled. Sonoelastography showed quadriceps hardness did not differ significantly between groups (p = 0.887) and a lower elastography ratio of quadriceps over subcutaneous tissue than the non-sarcopenia group (**Table 1**; **Figure 1**). Multivariate logistical regression analysis showed that the quadriceps to subcutaneous ratio, hardness and BMI could predict sarcopenia (p<0.05) (**Table 2**), while a lower BMI increased the risk of sarcopenia. The optimal quadriceps-to-subcutaneous tissue elastography ratio cut-off value was 0.885 (sensitivity 82.4%; specificity 66.7%).

**Table 1:** Baseline characteristics of the study population.

Variables	ESKD with sarcopenia (n=50)	ESKD non-sarcopenia (n=50)	*P-*value
Age (years)	63.02 ± 12.72	58.30 ± 14.93	0.140
Body mass index (kg/m^2^)	23.34 ± 1.95	27.89 ± 2.40	0.001
Gender (Female %)	22 (44%)	36 (72 %)	0.008
Ratio	0.71 ± 0.30	1.01 ± 0.59	0.002
Hardness(%)	72.03 ± 25.22	83.38 ± 27.09	0.887
Diabetes mellitus (%)	17 (34)	8 (16)	0.630
Hypertension (%)	32 (64)	29 (58)	0.682
Heart disease (%)	4 (8)	0 (0)	0.120
Liver disease (%)	6 (12)	0 (0)	0.007
Rheumatoid arthritis (%)	10(14.7)	16 (29.6)	0.074

**Table 2:** Multivariable analysis of factors associated with sarcopenia.

Variables	Regression coefficient	S.E.	Wald	P- value	OR (95%CI)
Age (years)	0.049	0.027	3.253	0.071	1.059 (0.996-1.107)
Body mass index (kg/m2)	-0.303	0.111	7.497	0.006	0.738 (0.594-0.917)
Gender	-1.146	0.835	0.031	0.861	0.864 (0.168-4.439)
Ratio	-2.777	1.029	7.281	0.007	0.062 (0.008-0.468)
Hardness(%)	-0.054	0.015	12.479	0.001	0.947 (0.919-0.976)
Diabetes mellitus	-0.047	0.846	0.003	0.955	0.954 (0.181-5.011)
Hypertension	-0.698	0.724	0.928	0.335	0.498 (0.120-2.058)
Heart disease	0.353	0.739	0.228	0.633	1.423 (0.334-6.061)
Pulmonary disease	0.192	1.218	0.025	0.875	1.212 (0.111-13.182)
Rheumatoid arthritis	-0.415	0.697	0.354	0.552	0.660 (0.168-2.589)

OR, odds ratio; SE, standard error

## Discussion

This study demonstrates that sonoelastography of the quadriceps muscle is a valuable and non-invasive tool for predicting sarcopenia in patients with end-stage kidney disease (ESKD). By utilizing the quadriceps-to-subcutaneous tissue elastography ratio and muscle hardness, we found that sonoelastography provides strong predictive power for sarcopenia. The quadriceps muscle plays a critical role in daily functional activities, and its structural and mechanical properties are closely linked to mobility and independence in older and chronically ill populations (24). Our findings establish a feasible protocol for obtaining reproducible sonoelastographic measurements of the quadriceps muscle.

Degenerative changes in the quadriceps muscle can arise from lipid infiltration, repetitive microtrauma, and hypoxia, leading to microscopic structural alterations. Although the clinical diagnosis can be challenging, both ultrasound and magnetic resonance imaging have proven useful in detecting muscle abnormalities (19, 21). While muscle rupture is rare in healthy individuals, degenerative processes from aging, fatty infiltration, steroid use, and sarcopenia can predispose the muscle to rupture under relatively low mechanical stress (22, 23). High-frequency ultrasound transducers allow detailed visualization of the quadriceps muscle due to its superficial location. When imaged perpendicularly, the muscle exhibits high echogenicity, with longitudinal collagen fibrils surrounded by a peritendinous sheath, visible as parallel echogenic lines on ultrasound. Ultrasound has been reported to have high sensitivity and specificity for detecting quadriceps muscle rupture (24). Sonoelastography extends these capabilities by providing information on tissue stiffness and mechanical integrity (14, 25). In this study, we applied compression elastography, which measures tissue strain in response to controlled pressure (26). After reclassification using EWGSOP2, 48 patients were identified as sarcopenic and 52 as non-sarcopenic. Although the sample distribution remained comparable to that under EWGSOP (2010), this consistency is attributable to the clinical characteristics of our advanced ESKD cohort, in which reduced muscle mass was almost invariably accompanied by impaired muscle strength. Consequently, patients classified as sarcopenic by EWGSOP (2010) largely overlapped with those meeting EWGSOP2 criteria, resulting in similar statistical outcomes. This reanalysis confirms that our findings are robust across contemporary diagnostic standards and supports the external validity of quadriceps sonoelastography as a predictor of sarcopenia.

Previous studies have shown that diseased muscle appear softer on sonoelastography compared to healthy ones, with color mapping revealing a transition from red (stiff) to green (soft) (27). Consistent with these findings, our sarcopenia group exhibited significantly reduced muscle stiffness and increased heterogeneity in elastographic patterns. Given that muscle quality is influenced by adipose and fibrous tissue infiltration, sonoelastography may provide indirect insights into muscle composition and functional capacity (28, 29).

To our knowledge, this is the first study to assess quadriceps muscle elasticity using sonoelastography in ESKD patients. The observed decrease in muscle stiffness among sarcopenic patients suggests that sonoelastography could enhance diagnostic accuracy for muscle quality assessment. Furthermore, our results showed that lower BMI was associated with sarcopenia, aligning with prior reports linking low BMI to increased long-term mortality risk. Although our study did not include mortality data, future longitudinal research is warranted to explore prognostic implications.

This study has limitations. First, the sample size was relatively small. Second, sonoelastography is highly operator-dependent, and we did not assess inter- or intra-observer variability. Lastly, standardized protocols for quadriceps muscle sonoelastography are currently lacking, limiting cross-study comparisons. This study did not include inflammatory markers (e.g., CRP, TNF-α, IL-6), nutritional indicators (albumin), or functional performance tests (grip strength, gait speed). These are important confounders. We also lacked longitudinal follow-up for falls, hospitalization, and mortality. Operator variability and medication effects (steroids, immunosuppressants) were not analyzed. Future studies should address these aspects.

In conclusion, sonoelastography is a reliable, accurate, and accessible imaging modality for evaluating muscle stiffness and quality. It holds potential for early detection of sarcopenia-related muscle changes, guiding preventive and therapeutic strategies in ESKD patients.

Sarcopenia was diagnosed according to the revised European Working Group on Sarcopenia in Older People (EWGSOP2, 2019), which emphasizes muscle strength as the principal determinant. Handgrip strength, gait speed, and appendicular skeletal muscle mass (ASM/height²) by DXA were assessed. Sarcopenia was defined as low muscle strength combined with low muscle mass, and severe sarcopenia was defined as low muscle strength, low muscle mass, and low gait speed.

According to EWGSOP2 (2019) criteria, 48 patients were classified as sarcopenic and 52 as non-sarcopenic. Baseline demographic and clinical characteristics were similar to the previous analysis. The sarcopenia group demonstrated significantly lower quadriceps-to-subcutaneous tissue elastography ratios compared with the control group. Multivariate logistic regression confirmed that lower elastography ratio, reduced muscle hardness, and lower BMI remained independent predictors of sarcopenia (p < 0.05). The optimal cut-off value for the quadriceps-to-subcutaneous ratio was 0.885 (sensitivity 82.4%; 1-specificity 33.3%).

## Data Availability

The raw data supporting the conclusions of this article will be made available by the authors, without undue reservation.
